# A pilot imaging mass cytometry study of cutaneous nerve-immune cell interactions in post-herpetic neuralgia

**DOI:** 10.3389/fimmu.2026.1813643

**Published:** 2026-07-08

**Authors:** Siddhesh N. Telang, Jackson F. Karrasch, Philip M. Finch, Stefanie S. Kogias, Thomas R. O’Neil, Anthony L. Cunningham, Andrew N. Harman, Peter D. Drummond, Paul J. Austin

**Affiliations:** 1 School of Medical Sciences, Faculty of Medicine and Health, The University of Sydney, Sydney, NSW, Australia; 2 Brain and Mind Centre, School of Medical Sciences, The University of Sydney, Sydney, NSW, Australia; 3 Centre for Virus Research, Westmead Institute for Medical Research, The University of Sydney, Sydney, NSW, Australia; 4 Perth Pain Management Centre, Perth, WA, Australia; 5 School of Psychology, Murdoch University, Perth, WA, Australia

**Keywords:** chronic neuropathic pain, imaging mass cytometry (IMC), langerhans cells (LC), macrophage, neuroimmune interactions, neuroimmunology, post-herpetic neuralgia (PHN), T cell

## Abstract

Post-herpetic neuralgia (PHN) is a chronic pain condition that persists after shingles and involves dermatome-specific allodynia, disability, and psychological comorbidities. Cutaneous neuroimmune dysregulation is increasingly linked to chronic pain, but the mechanisms underlying PHN remain unclear. This study is the first high-parameter imaging analysis of PHN-affected skin using imaging mass cytometry (IMC). Skin biopsies from PHN-affected dermatomes (*n* = 6) and healthy controls (*n* = 6) were stained with a 13-marker antibody panel targeting immune cells and nerve fibres. Immune cell densities and their spatial proximity to nerve fibres were quantified in a customised analysis pipeline. Langerhans cell density and skin-homing memory T cells were elevated in PHN-affected skin. There were trends towards higher T cells, infiltrating T helper type 1-like T cells, and T cell-nerve fibre interactions. While overall macrophage and DC subset numbers were unchanged, anti-inflammatory macrophage-nerve fibre interactions were significantly increased, but decreased across both DC subsets, suggesting selective spatial reorganisation of myeloid cell populations in the nerve fibre microenvironment. Nerve fibre density itself was unchanged. These findings suggest that persistent neuroimmune alterations may be involved in PHN pathophysiology. Our pilot study highlights the potential of IMC to reveal previously unrecognised cutaneous pathomechanisms that may contribute to PHN. Future IMC studies with larger sample sizes should be undertaken to guide the development of novel therapeutic strategies for PHN.

## Introduction

Reactivation of latent varicella zoster virus (VZV) in sensory ganglia (1) produces a painful dermatomal rash (shingles), and occasionally triggers pain that persists beyond three months after rash resolution (termed post-herpetic neuralgia; PHN) (2, 3). PHN is associated with profound physical disability and significant psychological comorbidities that reduce quality of life (4). The pathophysiology of PHN is not completely understood, but previous research implicates reduced intraepidermal nerve fibre (IENF) density (5, 6) and T cell infiltration into regions critical for pain perception, such as dorsal root ganglia (DRG), which contain the cell bodies of nociceptors (7, 8). Current treatment modalities focus on symptomatic relief rather than reversing underlying pathology, and although vaccination is highly effective at providing long-term protection from shingles and PHN (9–11), uptake varies globally due to limited awareness and concerns regarding efficacy and cost (12).

The immune system plays a critical role in the onset and maintenance of neuropathic pain conditions, including PHN (13, 14), with cutaneous neuroimmune interactions being key contributors (15). Langerhans cells (LC) and dendritic cells (DC) are specialised antigen-presenting cells in the skin that are strategically located to sample cutaneous antigens and stimulate early inflammatory responses to infectious pathogens (16, 17). LCs play a critical role in nociception in models of neuropathic pain (16, 18, 19) and regulate IENF density (IENFD) through direct nerve fibre interactions (20–22). VZV infection downregulates antigen presentation by DCs, impairing T cell activation and antiviral immunity (23), but direct effects on LC and DC interactions with nerve fibres have not been assessed in PHN. Macrophages contribute to neuropathic pain (13), but with variable correlation to pain severity (24, 25) and IENFD (26, 27), and their functional activation states are under-investigated (13). T cells are essential for antiviral immunity and contribute directly to neuropathic pain (28), with preclinical studies demonstrating that T helper type 1 (Th1)-biased responses enhance painful hypersensitivity (29, 30). Clinically, T cells infiltrate PHN-affected DRGs (7, 8, 31), and VZV-specific CD4^+^ T cells are elevated in PHN patient blood (32), but characterisation of cutaneous T cell-nerve fibre interactions in PHN is lacking (15).

Despite this growing evidence, neuroimmune interactions in PHN-affected skin remain underexplored, and no studies have used high-parameter spatial imaging to investigate these relationships. Imaging mass cytometry (IMC) enables the simultaneous visualisation of multiple cutaneous proteins *in situ* while preserving tissue architecture, providing unprecedented insight into neuroimmune dynamics (33). Here, we utilise IMC to quantify LC, DC, macrophage, and T cell abundance and examine their spatial proximities to nerve fibres in skin biopsies from PHN-affected dermatomes and from healthy controls. This study aims to advance our mechanistic understanding of PHN pathogenesis and to identify potential targets for future therapeutic intervention.

## Materials and methods

### Participant recruitment and tissue collection

Collection and processing of tissue specimens was performed under ethical approval from the Murdoch University Human Research Ethics Committee (Project No. 2011/233). All study participants provided informed written consent. Participant details are found in **Supplementary Table 1**.

Individuals diagnosed with post-herpetic neuralgia (PHN) were recruited from a small private pain clinic. Healthy control (HC) participants were volunteers with no relevant clinical diagnosis and did not undergo any clinical evaluation or intervention related to the study. Zamboni’s-fixed, paraffin-embedded (ZFPE) 3 mm skin biopsies were obtained from PHN-affected dermatomes (*n* = 6, 4 females, aged 64–83 years) and from HCs (*n* = 6, 3 females, aged 40–65 years) at Murdoch University under aseptic conditions. For the PHN cohort, skin biopsies were taken from the centre of the cutaneous region previously affected by the herpes zoster eruption and subsequently associated with the development of PHN. In a subset of PHN participants, paired contralateral unaffected skin biopsies were also obtained from the mirrored anatomical site relative to the PHN-affected dermatome (*n* = 3, 2 females, aged 64–72 years). For the HC cohort, skin biopsies were taken from the dorsal aspect of the hand or foot. All skin biopsies were performed following local anaesthesia infiltration with 1% lignocaine.

### Imaging mass cytometry

An optimised antibody panel targeting 13 cutaneous antigens was used to visualise immune, neural, and structural markers *in situ* (**Supplementary Table 2**). Experimental protocols and data acquisition were performed as previously described (34). Briefly, ZFPE skin sections (7 μm) were cut and mounted onto adhesive slides, air-dried, and baked overnight at 60°C. Sections were deparaffinised in xylene, rehydrated through a graded series of ethanol solutions, and washed in distilled water prior to heat induced antigen retrieval (pH 9 buffer, 95°C, 30 minutes). Routine TPBS washes were performed between each of the subsequent steps. Sections were blocked with 20% normal horse serum, followed by treatment with avidin/biotin blocking kit (Abcam, ab64212) and antibody diluent/block (Akoya Biosciences, ARD1001EA). A multiplex immunolabelling protocol was then applied: sections were incubated overnight at 4°C with three primary antibodies (mouse anti-human PGP9.5, rabbit anti-human Class III β-Tubulin (C3BT), and goat anti-human Langerin (Cy3 conjugate), followed by two secondary antibodies (anti-mouse IgG (Cy5 conjugate) and anti-rabbit IgG (biotin conjugate)) for one hour at room temperature. A panel of metal isotope-conjugated antibodies, which included a secondary antibody against Cy3 and two tertiary antibodies against Cy5 and biotin, was applied and left overnight at 4°C. Sections were fixed (4% paraformaldehyde), stained with DNA Intercalator-Ir (DVS Sciences, 201192A), rinsed, and air-dried. All immunolabelled sections were imaged using a Hyperion™ Imaging System (Standard BioTools). Regions of interest spanning the dermal-epidermal border were selected following optimisation of laser ablation parameters. Data (images) were acquired as.MCD files and stored for downstream analysis. Antibody specificity was validated based on signal localisation, exclusivity, morphology, and co-localisation with nuclear staining.

### Quantitative data analysis

A quantitative data analysis pipeline was adapted from the IMComplete-Workflow (https://github.com/CVR-MucosalImmunology/Image-Processing-Workflow) developed at the Centre for Virus Research, Westmead Institute for Medical Research. Metadata and multi-channel image stacks were extracted from.MCD files in Jupyter Notebook. A custom cell segmentation model was trained in Cellpose, then used for batch cell segmentation in Jupyter Notebook. For each multi-channel image stack, the epidermis and dermis were manually outlined with the Polygon Selection Tool in ImageJ. Binary masks of the epidermis and dermis were then created using the Mask(s) from ROI(s) plugin. Single-channel images of the neural markers PGP9.5 and C3BT were combined using the Image Calculator function to identify signal colocalisation. PGP9.5^+^C3BT^+^ pixels were then manually outlined using the Polygon Selection Tool and converted into a binary nerve fibre mask using the Mask(s) from ROI(s) plugin. Nerve fibre density was calculated by counting PGP9.5^+^C3BT^+^ pixels within the epidermis and dermis and standardising to 1,000,000 pixels (1 mm^2^) of epidermal (IENFD) and dermal (dermal nerve fibre density) area.

A customised CellProfiler workflow was used to measure single-cell marker expression, neighbourhood information, and nerve fibre proximity (distance of each cell to the nearest PGP9.5^+^C3BT^+^ pixel, in microns). The resulting output was used in RStudio to construct a dataframe containing all measured parameters. A SpatialExperiment object (SPE) was created from the dataframe, and raw marker values were arcsinh-transformed and min-max scaled (0–1). An.FCS file for each multi-channel image stack was generated from the SPE and imported into FlowJo, where a gating strategy, in conjunction with visual confirmation in Mantis Viewer (35), was used to identify cell populations of interest, which were then appended to the SPE metadata. Cell counts and nerve fibre proximity data were exported and standardised in Excel. For cell-nerve fibre proximity analyses, the distance of each cell to its closest nerve fibre was measured up to a spatial threshold of 40 μm. A 40 μm radius was used as a conservative operational upper limit for potential cell-nerve fibre spatial interactions, informed by prior *in vitro* modelling and experimental observations of short-range cytokine and chemokine-mediated cell communication (36, 37). This threshold should not be interpreted as a fixed physiological boundary, particularly in the epidermis where the dense architecture may restrict diffusion, but rather as a spatial criterion for identifying cell-nerve fibre proximity compatible with local paracrine signalling. The number of spatial interactions, i.e., the number of cells within this threshold, was then standardised to 1,000,000 pixels (1 mm^2^) of the appropriate tissue compartment. Data were plotted in GraphPad Prism.

### Statistics

Statistical analyses were performed in GraphPad Prism. All statistical analyses were exploratory in nature and, therefore, not corrected for multiple comparisons. Data distribution was assessed visually and using Shapiro-Wilk and Kolmogorov-Smirnov tests, recognising that formal normality testing has limited interpretability with very small sample sizes. If normality was satisfied, a Welch’s *t*-test was performed. If normality was violated, a Mann-Whitney U test was used. For comparisons between PHN-affected and contralateral unaffected biopsies, an unpaired approach was used due to incomplete pairing (only *n* = 3 paired contralateral unaffected biopsies). Welch’s *t*-test was selected to allow inclusion of all available samples while avoiding the assumption of equal variance. However, this approach does not explicitly account for within-subject pairing and, therefore, should be interpreted as exploratory. Across all comparisons, an α level of 0.05 was used to determine statistical significance.

## Results

A total of nine cell populations were identified and quantified using a hierarchical gating strategy based on protein expression: CD207^+^ (LC), CD207^+^HLA-DR^+^ (HLA-DR^+^ LC), CD3^+^ (T cell), CD3^+^CXCR3^+^ (infiltrating Th1-like T cell), CD3^+^CLA^+^ (skin-homing memory T cell), CD68^+^ (macrophage), CD68^+^CD206^+^ (anti-inflammatory macrophage), HLA-DR^+^CD68^-^CD206^+^ (CD206^+^ DC), and HLA-DR^+^CD68^-^CD206^-^ (CD206^-^ DC).

### Cutaneous nerve fibre density is unchanged in PHN

**Figure 1:**
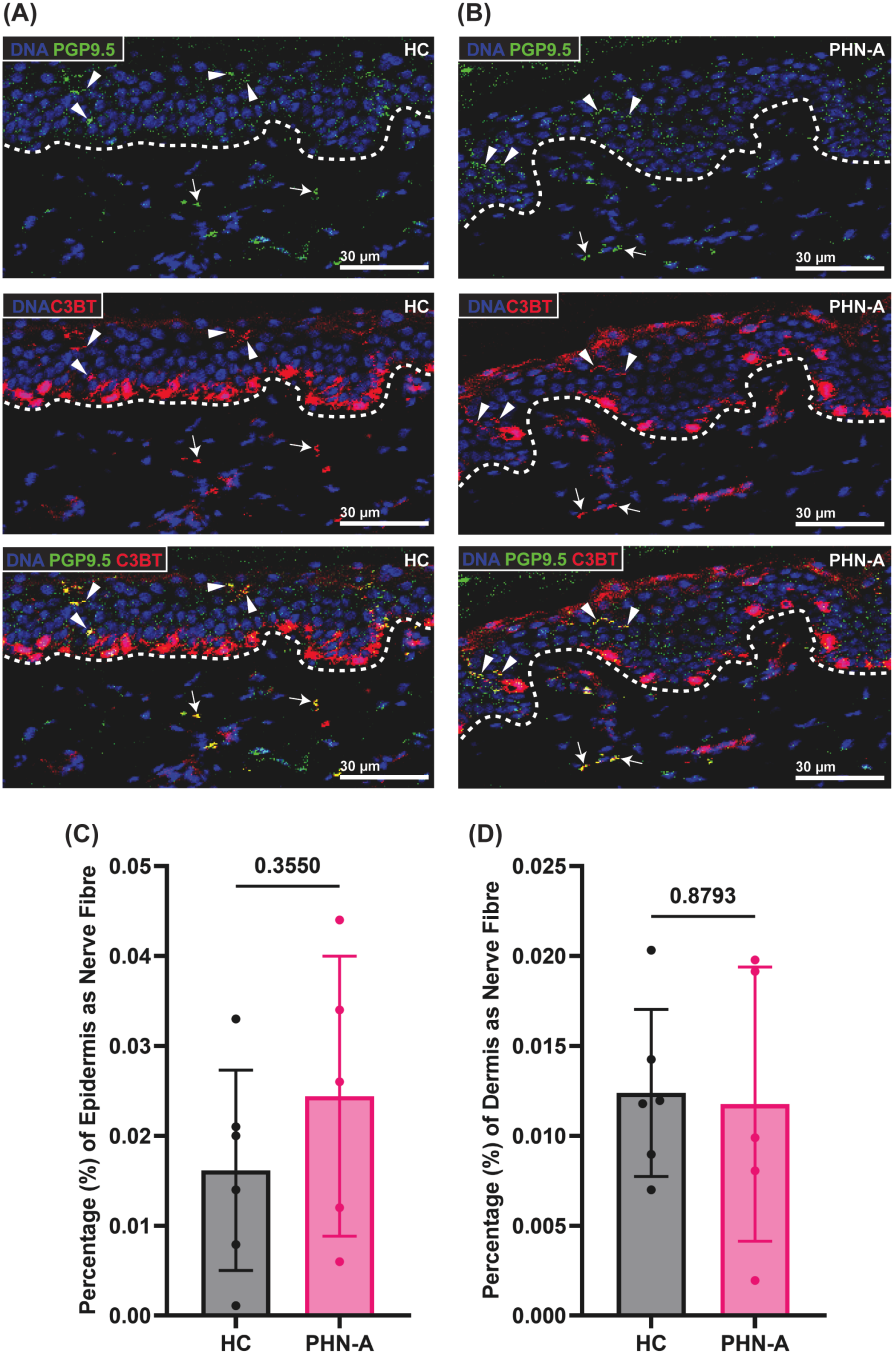
Nerve fibre density did not differ between skin biopsies from PHN-affected dermatomes and healthy controls. **(A, B)** Representative IMC images of PGP9.5^+^C3BT^+^ intraepidermal nerve fibres (white arrowheads) and dermal nerve fibres (white arrows) in PHN-affected (right) and healthy control (left) skin, with dermal-epidermal borders indicated by white dashed lines. **(C, D)** Column graphs comparing intraepidermal and dermal nerve fibre density in PHN-affected (*n* = 5) and healthy control (*n* = 6) skin. Data are presented as mean percentage of tissue compartment (epidermis or dermis) ± SD; Welch’s *t*-test. Statistical significance was set at *p* < 0.05. IMC, imaging mass cytometry; PHN, post-herpetic neuralgia; SD, standard deviation; PGP9.5, protein gene product 9.5; C3BT, class III β-tubulin; PHN-A, PHN-affected; HC, healthy control.

IMC detected epidermal and dermal nerve fibres in PHN-affected and HC skin (**Figures 1A, B**). However, their density was similar in both groups (**Figures 1C, D**, both *p* > 0.05).

### Increased langerhans cell abundance in PHN, but langerhans cell-nerve fibre proximity remains unchanged

**Figure 2:**
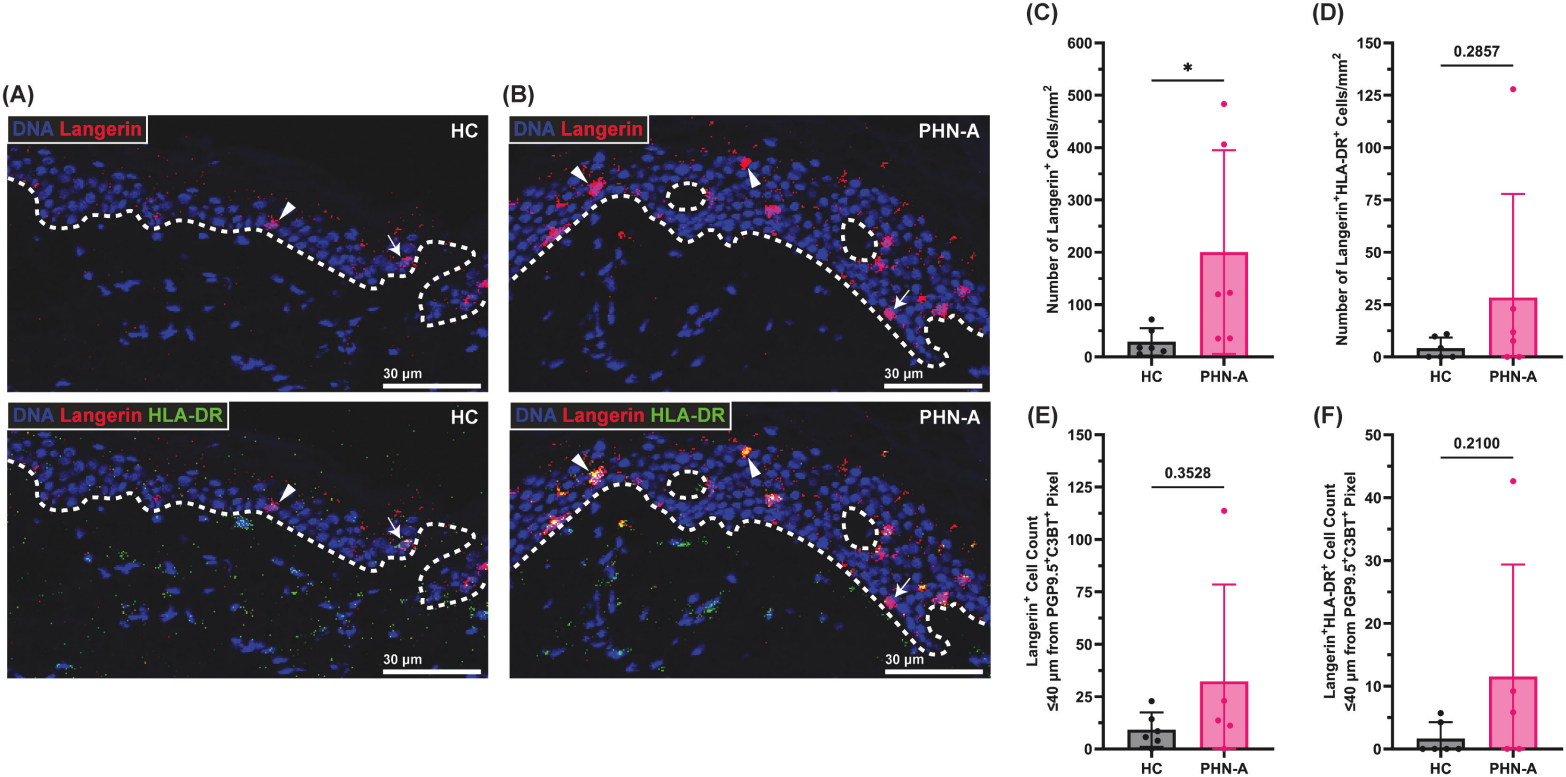
Skin biopsies from PHN-affected dermatomes exhibit increased Langerhans cell abundance, but not Langerhans cell-intraepidermal nerve fibre spatial interactions, compared to healthy controls. [**(A, B)**, top] Representative IMC images of epidermal Langerhans cells in PHN-affected (right) and healthy control (left) skin, with dermal-epidermal borders indicated by white dashed lines. (A-B, bottom) Langerhans cells lacking HLA-DR co-expression (Langerin^+^HLA-DR^-^, white arrowheads) and Langerhans cells co-expressing HLA-DR (Langerin^+^HLA-DR^+^, white arrows). **(C)** Column graph comparing Langerhans cell abundance in PHN-affected (*n* = 6) and healthy control (*n* = 6) skin. Data are presented as mean cells/mm^2^ ± SD; Mann-Whitney U test. **(D)** Column graph comparing HLA-DR^+^ Langerhans cell abundance in PHN-affected (*n* = 6) and healthy control (*n* = 6) skin. Data are presented as mean cells/mm^2^ ± SD; Mann-Whitney U test. **(E)** Column graph comparing the frequency of Langerhans cell-intraepidermal nerve fibre spatial interactions (0-40 μm) in PHN-affected (*n* = 5) and healthy control (*n* = 6) skin. Data are presented as mean interactions/mm^2^ ± SD; Mann-Whitney U test. **(F)** Column graph comparing the frequency of HLA-DR^+^ Langerhans cell-intraepidermal nerve fibre spatial interactions (0-40 μm) in PHN-affected (*n* = 5) and healthy control (*n* = 6) skin. Data are presented as mean interactions/mm^2^ ± SD; Mann-Whitney U test. Statistical significance was set at *p* < 0.05. IMC, imaging mass cytometry; PHN, post-herpetic neuralgia; SD, standard deviation; HLA-DR, human leukocyte antigen-DR; PGP9.5, protein gene product 9.5; C3BT, class III β-tubulin; PHN-A, PHN-affected; HC, healthy control.

The epidermis is known to contain both CD206^-^ LCs and CD206^+^ epidermal DCs (epi-DC) (38–41). All LCs express Langerin (CD207), as do a small proportion of epi-DCs (41). CD206^+^ epi-DCs were absent from our samples, whereas CD207^+^ LCs were present in PHN-affected and HC skin, some of which highly expressed the antigen presentation marker human leukocyte antigen-DR (HLA-DR) (**Figures 2A, B**). Total LC density was significantly higher in PHN-affected skin (**Figure 2C**; PHN-A: 200.30 ±194.60 cells/mm^2^ and HC: 28.78 ±26.11 cells/mm^2^, *p* = 0.0260), but HLA-DR^+^ LC density was similar in PHN-affected and HC skin (**Figure 2D**, *p* > 0.05). The frequency of LC-IENF spatial interactions, defined as an LC located ≤40 μm from its closest nerve fibre, was similar in PHN-affected and control skin (**Figure 2E**, *p* > 0.05). Likewise, the frequency of HLA-DR^+^ LC-IENF spatial interactions was unchanged (**Figure 2F**, *p* > 0.05).

### Increased CLA^+^ T lymphocyte abundance in PHN

**Figure 3:**
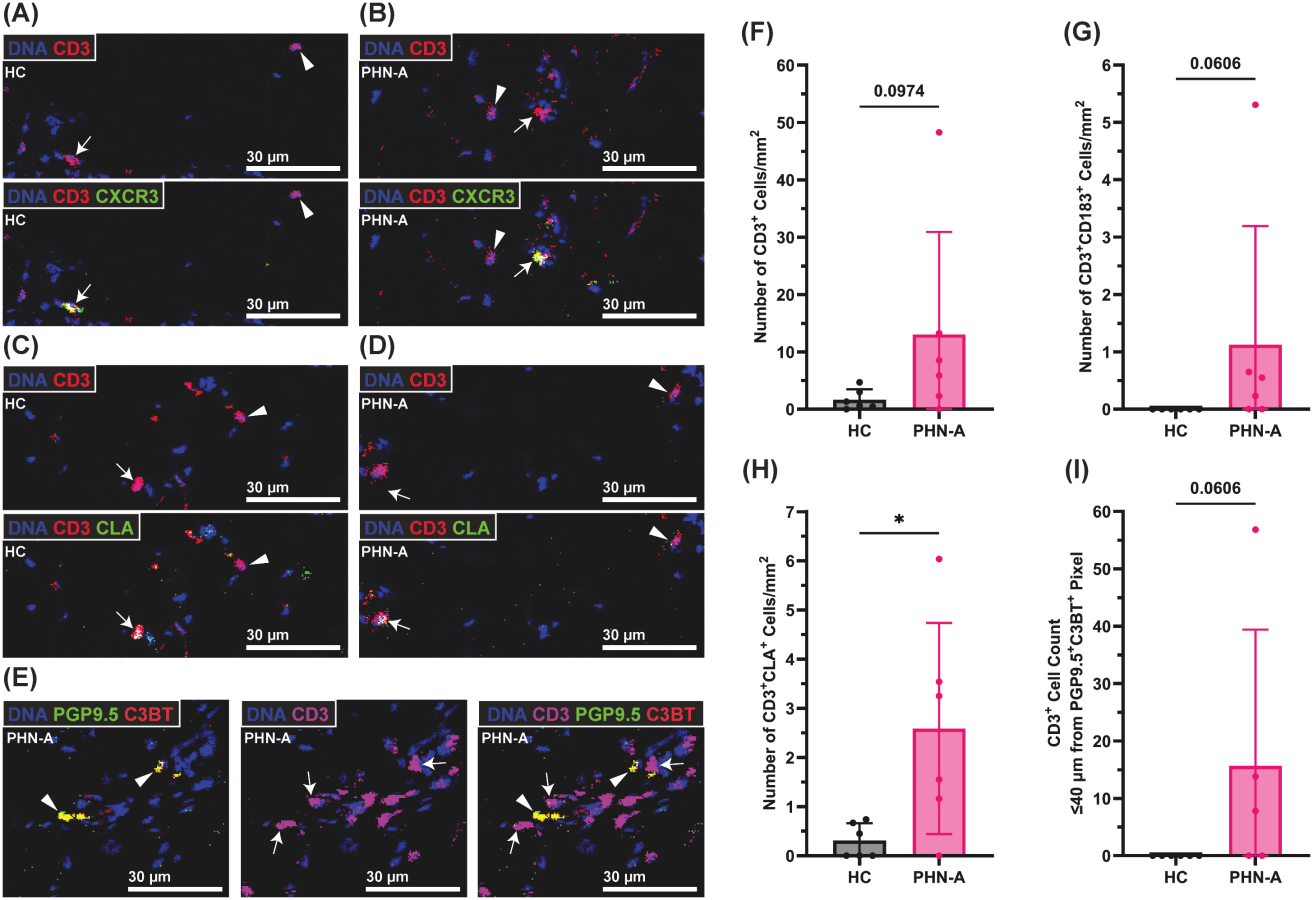
A subset of T cells co-expressing CLA was increased in skin biopsies from PHN-affected dermatomes relative to healthy controls. [**(A–D)**, top) Representative IMC images of dermal T cells in PHN-affected (right) and healthy control (left) skin. [**(A, B)**, bottom] T cells without CXCR3 co-expression (CD3^+^CXCR3^-^, white arrowheads) and T cells co-expressing CXCR3 (CD3^+^CXCR3^+^, white arrows). [**(C, D)**, bottom) T cells without CLA co-expression (CD3^+^CLA^-^, white arrowheads) and T cells co-expressing CLA (CD3^+^CLA^+^, white arrows). **(E)** Representative IMC images of dermal T cells in close proximity(≤40 μm) to dermal PGP9.5^+^C3BT^+^ nerve fibres in PHN-affected skin. **(F)** Column graph comparing T cell abundance in PHN-affected (*n* = 6) and healthy control (*n* = 6) skin. Data are presented as mean cells/mm^2^ ± SD; Mann-Whitney U test. **(G)** Column graph comparing CD183^+^ (CXCR3^+^) T cell abundance in PHN-affected (*n* = 6) and healthy control (*n* = 6) skin. Data are presented as mean cells/mm^2^ ± SD; Mann-Whitney U test. **(H)** Column graph comparing CLA^+^ T cell abundance in PHN-affected (*n* = 6) and healthy control (*n* = 6) skin. Data are presented as mean cells/mm^2^ ± SD; Welch’s *t*-test. **(I)** Column graph comparing the frequency of T cell-dermal nerve fibre spatial interactions (0-40 μm) in PHN-affected (*n* = 5) and healthy control (*n* = 6) skin. Data are presented as mean interactions/mm^2^ ± SD; Mann-Whitney U test. Statistical significance was set at *p* < 0.05. IMC, imaging mass cytometry; PHN, post-herpetic neuralgia; SD, standard deviation; CD183/CXCR3, C-X-C motif chemokine receptor type 3; CLA, cutaneous lymphocyte-associated antigen; PGP9.5, protein gene product 9.5; C3BT, class III β-tubulin; PHN-A, PHN-affected; HC, healthy control.

Dermal T cells, some co-expressing the inflammatory chemotaxis marker C-X-C motif chemokine receptor type 3 (CXCR3) or the skin-homing memory T cell marker cutaneous lymphocyte-associated antigen (CLA), were detected in PHN-affected and HC skin (**Figures 3A–D**). A number of dermal T cells were observed within 40 μm of dermal PGP9.5^+^C3BT^+^ nerve fibres in PHN-affected skin (**Figure 3E**). There was a trend towards increased total T cell density in PHN-affected skin (**Figure 3F**; PHN-A: 13.04 ±17.89 cells/mm^2^ and HC: 1.68 ±1.80 cells/mm^2^, *p* = 0.0974). Infiltrating Th1-like T cells were present in 4 of 6 (67%) PHN-affected specimens and zero of six (0%) HC specimens (**Figure 3G**). Thus, there was a trend for increased infiltrating Th1-like T cell abundance in PHN-affected skin (**Figure 3G**; PHN-A: 1.12 ±2.07 cells/mm^2^ and HC: 0.00 ±0.00 cells/mm^2^, *p* = 0.0606), and skin-homing memory T cell density was significantly higher in PHN-affected skin (**Figure 3H**; PHN-A: 2.59 ±2.15 cells/mm^2^ and HC: 0.31 ±0.35 cells/mm^2^, *p* = 0.0479). T cell-nerve fibre spatial interactions were present in 3 of 5 (60%) PHN-affected specimens and none of the six (0%) HC specimens (**Figure 3I**), with 15.69 ±23.73 interactions/mm^2^ in PHN-A and 0.00 ±0.00 interactions/mm^2^ in HC, which trended towards significance (*p* = 0.0606).

### No changes in macrophage or dendritic cell subset abundance, but altered CD206^+^ macrophage-, CD206^+^ DC-, and CD206^-^ DC-nerve fibre proximity, in PHN

**Figure 4:**
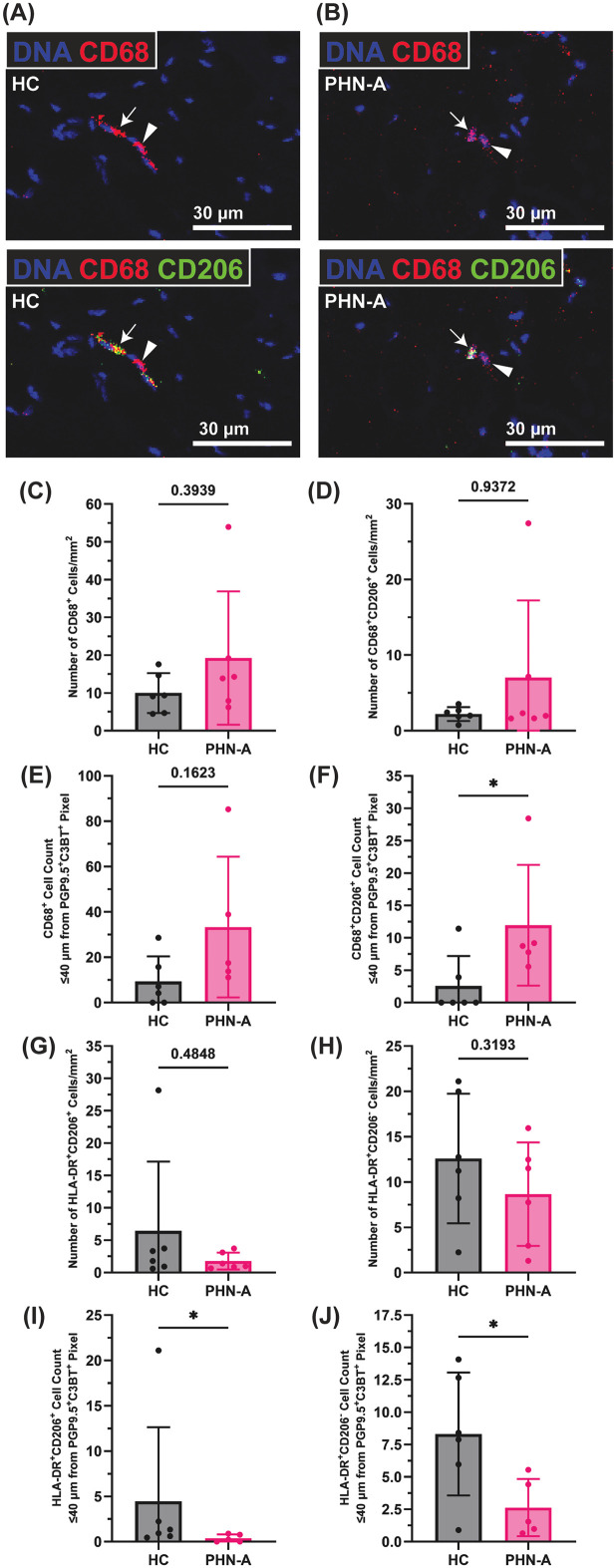
Increased CD206^+^ macrophage-dermal nerve fibre spatial interactions and reduced dendritic cell-dermal nerve fibre spatial interactions in PHN-affected dermatomes relative to healthy controls despite unchanged cell subset abundance. [**(A, B)**, top) Representative IMC images of dermal macrophages in healthy PHN-affected (right) and healthy control (left) skin. [**(A, B)**, bottom) Macrophages without CD206 co-expression (CD68^+^CD206^-^, white arrowheads) and macrophages co-expressing CD206 (CD68^+^CD206^+^, white arrows). **(C)** Column graph comparing macrophage abundance in PHN-affected (*n* = 6) and healthy control (*n* = 6) skin. Data are presented as mean cells/mm^2^ ± SD; Mann-Whitney U test. **(D)** Column graph comparing CD206^+^ macrophage abundance in PHN-affected (*n* = 6) and healthy control (*n* = 6) skin. Data are presented as mean cells/mm^2^ ± SD; Mann-Whitney U test. **(E)** Column graph comparing the frequency of macrophage-dermal nerve fibre spatial interactions (0-40 μm) in PHN-affected (*n* = 5) and healthy control (*n* = 6) skin. Data are presented as mean interactions/mm^2^ ± SD; Welch’s *t*-test. **(F)** Column graph comparing the frequency of CD206^+^ macrophage-dermal nerve fibre spatial interactions (0-40 μm) in PHN-affected (*n* = 5) and healthy control (*n* = 6) skin. Data are presented as mean interactions/mm^2^ ± SD; Mann-Whitney U test. **(G)** Column graph comparing CD206^+^ DC abundance in PHN-affected (*n* = 6) and healthy control (*n* = 6) skin. Data are presented as mean cells/mm^2^ ± SD; Mann-Whitney U test. **(H)** Column graph comparing CD206^-^ DC abundance in PHN-affected (*n* = 6) and healthy control (*n* = 6) skin. Data are presented as mean cells/mm^2^ ± SD; Welch’s *t*-test. **(I)** Column graph comparing the frequency of CD206^+^ DC-dermal nerve fibre spatial interactions (0-40 μm) in PHN-affected (*n* = 5) and healthy control (*n* = 6) skin. Data are presented as mean interactions/mm^2^ ± SD; Mann-Whitney U test. **(J)** Column graph comparing the frequency of CD206^-^ DC-dermal nerve fibre spatial interactions (0-40 μm) in PHN-affected (*n* = 5) and healthy control (*n* = 6) skin. Data are presented as mean interactions/mm^2^ ± SD; Welch’s *t*-test. Statistical significance was set at *p* < 0.05. IMC, imaging mass cytometry; PHN, post-herpetic neuralgia; SD, standard deviation; PGP9.5, protein gene product 9.5; C3BT, class III β-tubulin; PHN-A, PHN-affected; HC, healthy control.

Dermal macrophages were present in both PHN-affected and HC skin (**Figures 4A, B**). Total macrophage density was similar in PHN-affected and HC skin (**Figure 4C**, *p* > 0.05). Moreover, the abundance of anti-inflammatory macrophages was unchanged in PHN-affected skin (**Figure 4D**, *p* > 0.05). No significant differences in the frequency of macrophage-nerve fibre spatial interactions were observed (**Figure 4E**, *p* > 0.05). However, the frequency of anti-inflammatory macrophage-nerve fibre spatial interactions was increased in PHN-affected skin (**Figure 4F**; PHN-A: 11.94 ±9.32 interactions/mm^2^ and HC: 2.56 ±4.62 interactions/mm^2^, *p* = 0.0390). No significant differences in CD206^+^ DC density or CD206^-^ DC density were found (**Figures 4G, H**, both *p* > 0.05). However, frequencies of CD206^+^ DC- and CD206^-^ DC-nerve fibre spatial interactions were decreased in PHN-affected skin (**Figures 4I, J**; CD206^+^ DC, PHN-A: 0.38 ±0.42 interactions/mm^2^ and CD206^+^ DC, HC: 4.44 ±8.19 interactions/mm^2^, *p* = 0.0498; CD206^-^ DC, PHN-A: 2.63 ±2.21 interactions/mm^2^ and CD206^-^ DC, HC: 8.32 ±4.75 interactions/mm^2^, *p* = 0.0335).

### Nerve fibre density parameters and immune cell population abundance are similar between PHN-affected dermatomes and paired contralateral unaffected biopsy sites

No significant differences were identified for IENFD, dermal nerve fibre density, or any immune cell population between PHN-affected and contralateral unaffected skin (**Supplementary Figure 1**, both *p* > 0.05; **Supplementary Figure 2**, all *p* > 0.05).

## Discussion

This is the first high-parameter spatial imaging study of neuroimmune interactions in PHN-affected skin using IMC, highlighting the potential of this technology. By combining multiplexed *in situ* immunophenotyping with spatial proximity analyses, we investigated immune cell abundance and immune cell-nerve fibre interactions in skin biopsies from PHN-affected dermatomes and healthy controls. This small pilot study found unchanged epidermal and dermal nerve fibre density, but we observed increased LC and CLA^+^ T cell abundance and anti-inflammatory macrophage-nerve fibre interactions in PHN-affected skin compared to healthy control skin.

Previous studies have shown that IENFD is lower in PHN-affected dermatomes relative to contralateral unaffected and distant unaffected sites, but they do not compare to HCs (5, 6, 42–45), whereas we found no change relative to healthy skin. The largest of these studies, by Fetell et al., investigated 294 PHN patients and found an average reduction in IENFD of 20%, which was far more pronounced in those aged 70 years or older (5). Our findings further contrast with prior investigations that report loss of dermal myelinated nerve fibres in PHN-affected skin (43, 45). These discrepancies are likely due to the small sample size in our pilot study, as well as variations in biopsy location, disease stage, and methodology (e.g. tissue section thickness, imaging technique, controls, and quantification).

Here, epidermal LC density in PHN-affected skin was increased, though there was only a trend towards increased LC-IENF interactions with high individual variability. LC expansion is consistent with their well-established involvement in cutaneous antigen presentation (15, 16) and neurogenic inflammation (46–48) and likely reflects heightened immune system activation in PHN-affected skin. This finding differs from prior studies, which report no changes in LC abundance in disease-affected dermatomes from people with shingles who later developed PHN relative to those with shingles alone (44, 49). However, these studies are limited by the lack of a HC comparison group. LC expansion may sensitise peripheral nociceptors indirectly by initiating an adaptive immune response that drives pro-nociceptive inflammation (16, 50). Future studies should seek to clarify specific LC-mediated neuroimmune interactions, including whether LCs with distinct activation states–such as mature immunogenic (e.g., CD83^+^ and/or CD86^+^) (51) or immunoregulatory (e.g., PD-L1^+^) (52, 53) phenotypes–differentially contribute to PHN.

A significant increase in CLA^+^ T cell abundance was observed in PHN-affected skin, with trends towards elevated CXCR3^+^ T cells and increased T cell-nerve fibre interactions. T cells are strongly implicated in PHN through persistent activation and cytokine production (32, 54–59), although evidence for skin-specific involvement is mixed (60). Shingles rash sites exhibit increased VZV-specific CD4^+^ and CD8^+^ resident memory T cells (TRM) that persist long-term following rash resolution (61). PHN-affected skin in the elderly also contains increased FOXP3^+^ regulatory T cells and enhanced inhibitory receptor expression on VZV-specific CD4^+^ T cells (62), indicating localised suppression of antiviral immunity. However, other studies fail to find any evidence of dermal T cell infiltration in PHN-affected dermatomes (44) and, interestingly, one report concluded that individuals who developed PHN exhibited fewer infiltrating T lymphocytes in their shingles lesions than individuals whose shingles resolved (63). Nonetheless, VZV preferentially infects CLA^+^ memory T cells (64), which could facilitate T cell-mediated viral spread in the skin, consistent with the elevated CLA^+^ T cell abundance observed herein.

CLA is expressed across multiple memory T cell subsets, including both CD4^+^ and CD8^+^ TRMs (65, 66). Despite our best efforts, markers for helper, cytotoxic, and memory T cell subsets could not be optimised in Zamboni’s-fixed, paraffin-embedded skin using IMC (e.g., CD4, CD8, CD45RO, and CD69) (34), precluding definitive classification of CLA^+^ T cells as CD4^+^ or CD8^+^ TRMs. As dermal memory T cells are predominantly CD4^+^ (67, 68), CLA^+^ T cells identified here most likely represent a largely CD4^+^ skin-homing memory T cell population that includes a subset of bona fide TRMs. Given the long-term persistence of VZV-specific CD4^+^ TRMs (61), these cells are most plausibly retained from the resolved shingles rash rather than newly recruited during PHN. The persistence of these CLA^+^ T cells after an acute episode of shingles shows similarities to those persisting in skin after an acute episode of human genital herpes (69). In that study, Koelle et al. demonstrated high expression of CLA on circulating herpes simplex virus type 2 (HSV-2)-specific CD8^+^ T cells, which were also enriched in HSV-2 skin lesions, supporting a model of recruitment and local persistence following acute infection. A similar mechanism may also be relevant to herpes simplex virus type 1 (HSV-1)-associated herpes labialis, as HSV-1-specific CD8^+^ T cells can upregulate CLA following antigen exposure, although subsequent recruitment to, and persistence within, skin is unclear (70).

Total CD3^+^ T cell and CXCR3^+^ T cell densities were also numerically increased, though not statistically significant, suggesting possible involvement of additional T cell subsets. CXCR3 is a chemokine receptor that promotes migration to inflamed tissues and is associated with pro-nociceptive Th1 cells (71, 72), implicating these cells in PHN-related persistent pain. While T cell-nerve fibre proximity did not reach statistical significance, the absence of T cells within 40 μm of nerves in healthy skin indicates that neuroimmune interactions in PHN warrant further investigation. Expanded IMC panels or alternative high-parameter imaging platforms are required to define T cell functional states and clarify neuroimmune mechanisms relevant to PHN.

Macrophage abundance was unchanged in PHN-affected skin, but anti-inflammatory macrophage-nerve fibre interactions were significantly increased. Macrophages are known to contribute to chronic pain states through peripheral sensitisation (73), altered neuroimmune interactions (30, 74), and their phenotypic diversity (13). Although cutaneous macrophage involvement in PHN is unclear, elevated interleukin-6, which is secreted by macrophages, in serum from people with shingles (63) and macrophage infiltration in postmortem PHN-affected ipsilateral spinal cord (75) suggest a role for macrophages in the establishment of chronic pain. Preclinical studies show that macrophage-nociceptor crosstalk modulates pain sensitivity (76) and that bidirectional communication with peptidergic nociceptors can both drive and resolve pathological pain (77–79). Macrophages can adopt a pro-repair phenotype to promote tissue healing (79–81), and given that CD206 is expressed on anti-inflammatory macrophages (82, 83), increased CD206^+^ macrophage-nerve fibre interactions may reflect a compensatory pro-resolving mechanism in PHN. These alterations were not uniform across dermal myeloid cell populations, as CD206^+^ DC and CD206^-^ DC abundance were unchanged, but nerve fibre interactions were reduced for both subsets in PHN-affected skin. Together, these findings support a model of selective spatial reorganisation of dermal myeloid cells relative to dermal nerve fibres in PHN (13), rather than a general increase in dermal myeloid cell abundance.

There were no significant differences in IENFD or dermal nerve fibre density between PHN-affected and contralateral unaffected skin, but we did observe a trend towards a reduction in both parameters in PHN-affected skin, which is consistent with data previously reported (5). No significant differences were found for immune cell population densities between PHN-affected and contralateral unaffected skin, although LC, T cell and macrophage numbers trended higher in PHN-affected sites relative to contralateral unaffected. A larger cohort of contralateral unaffected biopsies should be used to investigate if immune cell numbers are increased relative to healthy controls.

Key limitations of the present pilot study include the small sample size and choice of controls (i.e., *n* = 6 PHN-affected dermatome biopsies, *n* = 3 contralateral unaffected biopsies, and *n* = 6 imperfectly age- and site-matched healthy control biopsies), incomplete clinical data (e.g., medications and comorbidities), and constraints imposed by the limited IMC antibody panel. Future studies should confirm these findings by incorporating larger cohorts, both contralateral unaffected and healthy controls, expanded phenotyping, and longitudinal sampling to track neuroimmune dynamics in PHN. By utilising IMC, our pilot study provides preliminary evidence of elevated LC and skin-homing CLA^+^ memory T cell abundance, as well as increased anti-inflammatory macrophage-nerve fibre, and reduced DC-nerve fibre spatial interactions, in PHN-affected skin. These findings suggest that persistent LC and T cell activation and reorganisation of dermal myeloid subsets may be contributors to PHN pathophysiology. Moreover, our data support the use of IMC as an investigative tool for examining cutaneous neuroimmune interactions in PHN and for informing future studies that explore its pathomechanisms and potential therapeutic avenues.

## Data Availability

The original contributions presented in the study are included in the article/**Supplementary Material**, further inquiries can be directed to the corresponding author/s.

## Supplementary material

Supplementary Table 1 Study participant information. Details of all study participants, including sex, age, time since shingles onset, and location of skin biopsy. Symptoms experienced in the PHN-affected dermatome for people with PHN are also described. Not applicable is denoted as “N/A”.

Supplementary Table 2 Antibodies used for imaging mass cytometry. The host species, reactivity, target antigen, marker specificity, conjugate (if applicable), working concentration, manufacturer, catalogue number, and clone are outlined for each antibody. The panel was designed to target nerve fibres (PGP9.5 and Class III β-Tubulin), various immune cell populations (e.g., CD68 and CD3), and structural markers (e.g., α-SMA and Ki67). Antibodies conjugated to heavy metal isotopes (e.g., 89Y, 149Sm, and 152Sm) were detected by IMC to visualise cutaneous proteins *in situ*. Not applicable is denoted as “N/A”.

Supplementary Figure 1 No significant differences in nerve fibre density between PHN-affected dermatomes and contralateral unaffected biopsy sites. (A-B) Column graphs comparing intraepidermal and dermal nerve fibre density in PHN-affected (*n* = 5) and paired contralateral unaffected (*n* = 3) skin. Data are presented as mean percentage of tissue compartment (epidermis or dermis) with lines connecting paired samples from the same participant; Welch’s *t*-test. Statistical significance was set at *p* < 0.05. Abbreviations: PHN, post-herpetic neuralgia; PHN-A, PHN-affected; PHN-CU, contralateral unaffected.

Supplementary Figure 2 No significant differences in immune cell population densities between PHN-affected dermatomes and paired contralateral unaffected skin **(A–I)** Column graphs comparing Langerhans cell **(A)**, HLA-DR^+^ Langerhans cell **(B)**, T cell **(C)**, CD183^+^ (CXCR3^+^) T cell **(D)**, CLA^+^ T cell **(E)**, macrophage **(F)**, CD206^+^ macrophage **(G)**, CD206^+^ DC **(H)**, and CD206^-^ DC **(I)** abundance in PHN-affected (*n* = 5) and paired contralateral unaffected (*n* = 3) skin. Data are presented as mean cells/mm^2^ with lines connecting paired samples from the same participant; Welch’s *t*-test. Statistical significance was set at *p* < 0.05. Abbreviations: PHN, post-herpetic neuralgia; HLA-DR, human leukocyte antigen-DR; CD183/CXCR3, C-X-C motif chemokine receptor type 3; CLA, cutaneous lymphocyte-associated antigen; PHN-A, PHN-affected; PHN-CU, contralateral unaffected.
