# Supplementary material for: A pilot imaging mass cytometry study of cutaneous nerve-immune cell interactions in post-herpetic neuralgia
Source: Front Immunol. 2026 Jul 8;17:1813643. doi: 10.3389/fimmu.2026.1813643 (PMC13388089; doi:10.3389/fimmu.2026.1813643)
Supplement: Supplementary Table 1 — Study participant information. Details of all study participants, including sex, age, time since shingles onset, and location of skin biopsy. Symptoms experienced in the PHN-affected dermatome for people with PHN are also described. Not applicable is denoted as “N/A”. [file SupplementaryFile1.docx]

**Supplementary Table 1. Study participant information.** Details of all study participants, including sex, age, time since shingles onset, and location of skin biopsy. Symptoms experienced in the PHN-affected dermatome for people with PHN are also described. Not applicable is denoted as “N/A”.

| Participant ID | Biopsy Location | Sex | Age | Time Since Shingles Onset (Months, Approx.) | Symptoms (PHN-Affected Dermatome) |
| --- | --- | --- | --- | --- | --- |
| PHN 1 | Left Upper Back | Female | 64 | 84 | Allodynia and hyperaesthesia to thermal and mechanical stimuli |
| PHN 2 | Right Upper Back | Female | 83 | 3.5 | Allodynia to thermal and mechanical stimuli |
| PHN 3 | Right Thorax | Male | 79 | 5.9 | Allodynia, followed by loss of sensation to thermal and mechanical stimuli |
| PHN 4 | Left Upper Back | Male | 72 | 23.9 | Allodynia to mechanical stimuli and loss of sensation to thermal stimuli |
| PHN 5 | Right Collar | Female | 75 | 7.5 | Loss of sensation |
| PHN 6 | Left Foot (Dorsal) | Female | 69 | 45.2 | Allodynia and hyperaesthesia to thermal and mechanical stimuli |
| HC 1 | Right Foot | Female | 57 | N/A | N/A |
| HC 2 | Left Hand | Male | 58 | N/A | N/A |
| HC 3 | Left Foot | Female | 40 | N/A | N/A |
| HC 4 | Left Hand | Female | 56 | N/A | N/A |
| HC 5 | Left Foot | Male | 60 | N/A | N/A |
| HC 6 | Left Hand | Male | 65 | N/A | N/A |

**Supplementary Table 2. Antibodies used for imaging mass cytometry.** The host species, reactivity, target antigen, marker specificity, conjugate (if applicable), working concentration, manufacturer, catalogue number, and clone are outlined for each antibody. The panel was designed to target nerve fibres (PGP9.5 and Class III β-Tubulin), various immune cell populations (e.g., CD68 and CD3), and structural markers (e.g., α-SMA and Ki67). Antibodies conjugated to heavy metal isotopes (e.g., 89Y, 149Sm, and 152Sm) were detected by IMC to visualise cutaneous proteins in situ. Not applicable is denoted as “N/A”.

| Host Species | Reactivity | Antigen | Marker | Conjugate | Manufacturer | Catalogue Number | Clone | Working Concentration (μg/mL) |
| --- | --- | --- | --- | --- | --- | --- | --- | --- |
| Mouse | X-Reactive | α-SMA | Blood Vessels | 89Y | R&D Systems | RDSMAB1420 | 1A4 | 4 |
| Mouse | X-Reactive | CLA | Skin-Homing Memory T Cells | 149Sm | BioLegend | 321302 | HECA-452 | 5.5 |
| Mouse | X-Reactive | Cyanine 3 | 2° | 152Sm | Santa Cruz Biotechnology | SC-166894 | A-6 | 3 |
| Mouse | Human | CD68 | Macrophages | 153Eu | BioLegend | 916104 | KPI | 1.5 |
| Mouse | Human | CD163 | M2 Macrophages | 154Sm | Novus Biologicals | NOVNB11040698 | EDHU-1 | 1.5 |
| Mouse | Human | CD183 (CXCR3) | Pro-Inflammatory Cytokine Receptor, Th1 T Cells | 156Gd | Abcam | ab64714 | 49801 | 0.5 |
| Sheep | Human | FXIIIa | Macrophages, Dendritic Cells, Fibroblasts | 161Dy | Affinity Biologicals | SAF13A-AP | Polyclonal | 0.25 |
| Mouse | X-Reactive | Ki67 | Cell Proliferation | 162Dy | BD Biosciences | 556003 | Polyclonal | 2 |
| Mouse | X-Reactive | Biotin | 3° | 164Dy | BioLegend | 409002 | 1D4-C5 | 0.5 |
| Mouse | X-Reactive | Cyanine 5 | 3° | 169Tm | Sigma-Aldrich | C1117 | CY5-15 | 1 |
| Rabbit | Human | CD3 | T Cells | 170Er | Agilent Technologies | A045229-2 | Polyclonal | 2 |
| Mouse | Human | CD206 | Anti-Inflammatory Macrophages and Dendritic Cells | 172Yb | R&D Systems | MAB25341 | 685645 | 2 |
| Rabbit | Human | HLA-DR | Antigen Presentation | 174Yb | Abcam | ab215985 | EPR3692 | 0.5 |
| N/A | N/A | DNA | Cell Nuclei | 191Ir | DVS Sciences | 201192A | N/A | 0.3125 μM |
| N/A | N/A | DNA | Cell Nuclei | 193Ir | DVS Sciences | 201192A | N/A | 0.3125 μM |
| Mouse | Human | PGP9.5 | Pan-Neuronal | N/A | Bio-Rad | 7863-1004 | 31A3 | 2.5 |
| Rabbit | X-Reactive | Class III β-Tubulin | Pan Neuronal, Melanocytes | N/A | Abcam | ab221935 | EP1569Y | 2 |
| Goat | Human | Langerin | Langerhans Cells | Cyanine 3 | R&D Systems | AF2088 | Polyclonal | 4 |
| Donkey | Mouse | Mouse IgG | 2° | Cyanine 5 | Jackson Immunoresearch | 715-175-151 | IgG | 2 |
| Donkey | Rabbit | Rabbit IgG | 2° | Biotin | Jackson Immunoresearch | 711-065-152 | IgG | 2 |


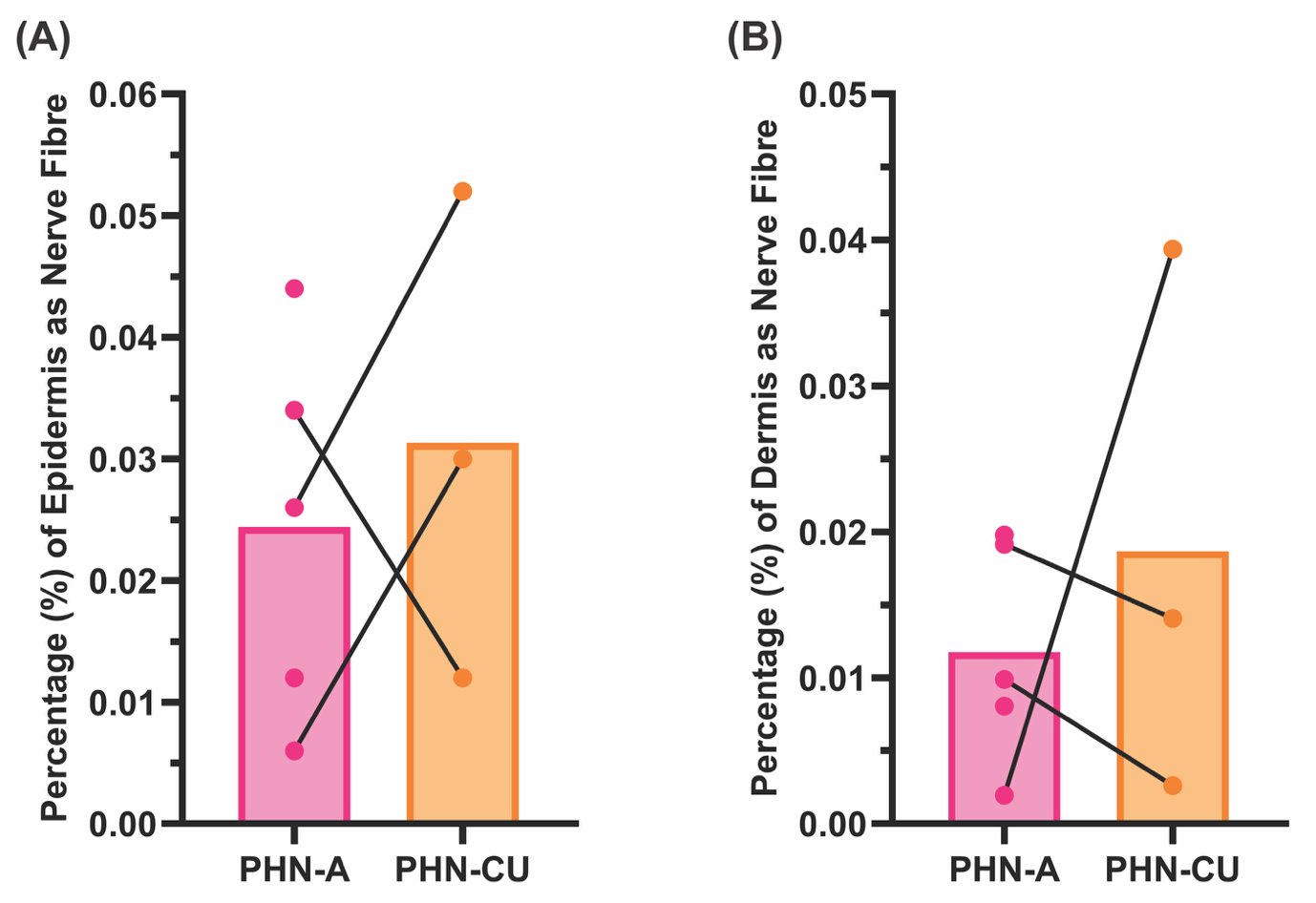


**Supplementary Figure 1.** **No significant differences in nerve fibre density between PHN-affected dermatomes and contralateral unaffected biopsy sites.** (A-B) Column graphs comparing intraepidermal and dermal nerve fibre density in PHN-affected (*n* = 5) and paired contralateral unaffected (*n* = 3) skin. Data are presented as mean percentage of tissue compartment (epidermis or dermis) with lines connecting paired samples from the same participant; Welch’s *t*-test. Statistical significance was set at *p* < 0.05. Abbreviations: PHN, post-herpetic neuralgia; PHN-A, PHN-affected; PHN-CU, contralateral unaffected.


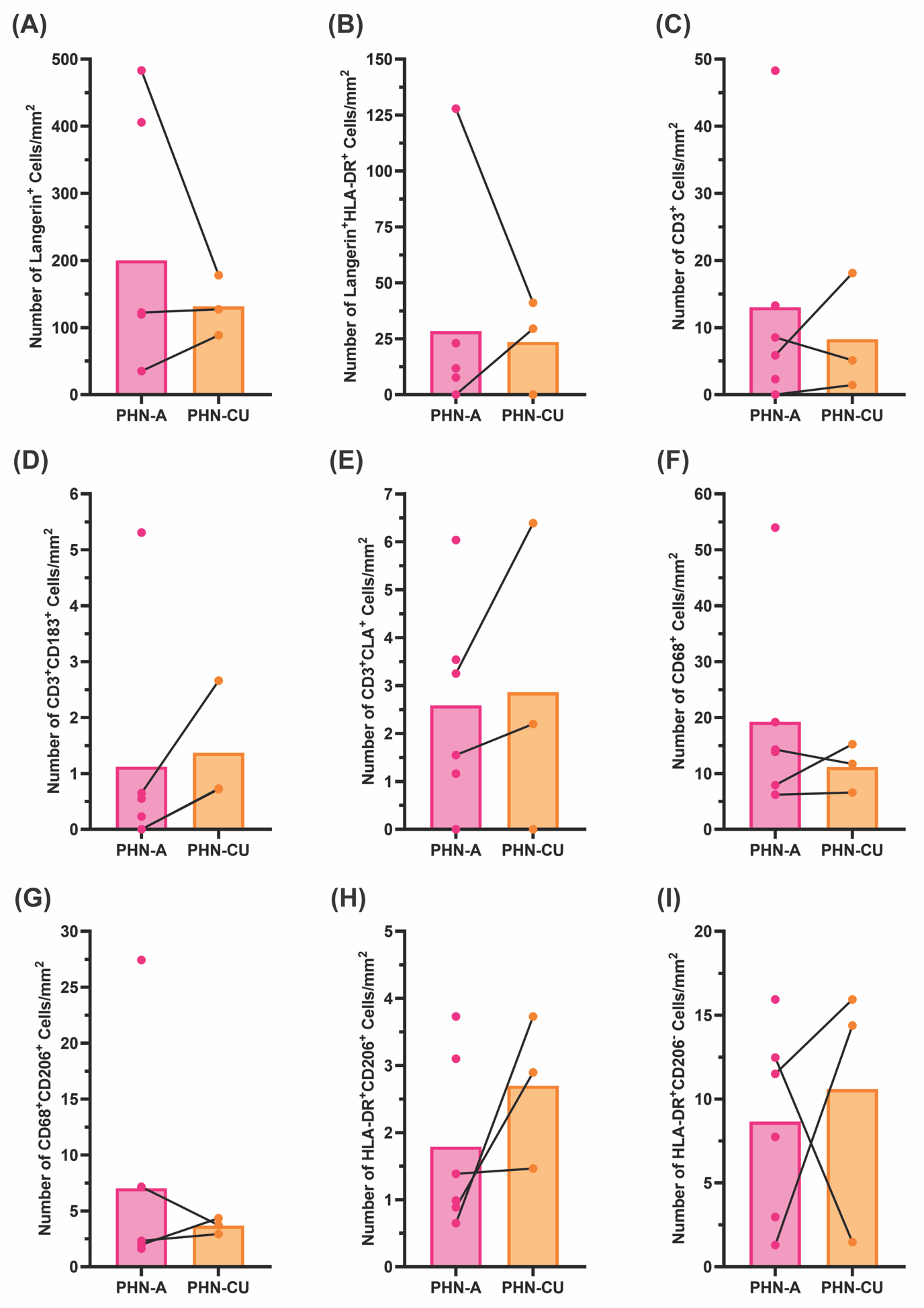


**Supplementary Figure 2.** **No significant differences in immune cell population densities between PHN-affected dermatomes and paired contralateral unaffected skin.** (A-I) Column graphs comparing Langerhans cell (A), HLA-DR^+^ Langerhans cell (B), T cell (C), CD183^+^ (CXCR3^+^) T cell (D), CLA^+^ T cell (E), macrophage (F), CD206^+^ macrophage (G), CD206^+^ DC (H), and CD206^-^ DC (I) abundance in PHN-affected (*n* = 5) and paired contralateral unaffected (*n* = 3) skin. Data are presented as mean cells/mm^2^ with lines connecting paired samples from the same participant; Welch’s *t*-test. Statistical significance was set at *p* < 0.05. Abbreviations: PHN, post-herpetic neuralgia; HLA-DR, human leukocyte antigen-DR; CD183/CXCR3, C-X-C motif chemokine receptor type 3; CLA, cutaneous lymphocyte-associated antigen; PHN-A, PHN-affected; PHN-CU, contralateral unaffected.
